# Adulthood personality correlates of childhood adversity

**DOI:** 10.3389/fpsyg.2014.01357

**Published:** 2014-11-21

**Authors:** Charles S. Carver, Sheri L. Johnson, Michael E. McCullough, Daniel E. Forster, Jutta Joormann

**Affiliations:** ^1^Department of Psychology, University of MiamiCoral Gables, FL, USA; ^2^Center for Advanced Study in the Behavioral SciencesStanford, CA, USA; ^3^Department of Psychology, University of CaliforniaBerkeley, Berkeley, CA, USA; ^4^Department of Psychology, Yale UniversityNew Haven, CT, USA

**Keywords:** adversity, pride, aggression, agreeableness, interdependence, social connection, extrinsic focus

## Abstract

**Objective:** Childhood adversity has been linked to internalizing and externalizing disorders and personality disorders in adulthood. This study extends that research by examining several personality measures as correlates of childhood adversity.

**Method:** In a college sample self-reports were collected of childhood adversity, several scales relating to personality, and current depression symptoms as a control variable. The personality-related scales were reduced to four latent variables, which we termed anger/aggression, extrinsic focus, agreeableness, and engagement.

**Results:** Controlling for concurrent depressive symptoms and gender, higher levels of reported childhood adversity related to lower agreeableness and to higher anger/aggression and extrinsic focus.

**Conclusions:** Findings suggest that early adversity is linked to personality variables relevant to the building of social connection.

## INTRODUCTION

What are the long-term consequences of adversity early in life? Early life adversity has been linked to a wide range of internalizing and externalizing psychopathologies in adulthood (Kessler et al., 1997; Anda et al., 2006; Gilbert et al., 2009; Evans et al., 2013; Evans and Cassells, 2014), including diagnosable personality disorders (Luntz and Widom, 1994; Battle et al., 2004), and subclinical features of personality disorders (Luntz and Widom, 1994; Meyer and Carver, 2000; Gibb et al., 2001; Carr and Francis, 2009). Researchers interested in psychological disorder have extensively studied associations with early adversity (assessed in various ways), but less is known about associations with normal personality. Are more wide-ranging, non-clinical aspects of personality associated with early adversity? The study reported here is a step at addressing this question.

A useful framework for considering the question is that of Belsky et al. (1991) and Belsky (2012), an evolutionary view which posits that children use early life experience to create a personal model of the availability and predictability of resources, the trustworthiness of other people, and the nature of interpersonal relations—enduring versus disposable. From this perspective, early adversity fosters expectations that resources will be unavailable and other people will be untrustworthy, yielding an opportunistic and even exploitive orientation to relationships (Belsky, 2012). Thus, early adversity may promote a range of social and behavioral tendencies, including aggressiveness, devaluation of social relations (low agreeableness), and an orientation to extrinsic incentives over social ones.

Another framework bearing on this question is “life history” theory (Kaplan and Gangestad, 2005), which deals with allocations of energy and time for various life tasks (e.g., reproduction, survival and body maintenance, growth). This view suggests that humans have mechanisms that are sensitive to factors in the environment which indicate variation in optimal allocation patterns (Del Giudice, 2014). Life history strategies are often discussed in terms of a fast–slow continuum. A fast life history orientation is one that favors short-term gains over longer-term benefits; a slow life history orientation takes the opposite pattern. Life history orientations (which are not considered to be conscious or intentional) are believed to be affected by variables such as availability of resources and environmental predictability early in life (Del Giudice, 2014). Consistent wit this view, early adversity does seem to foster an implicit expectation that life is short and must be lived quickly (Brumbach et al., 2009; Ellis et al., 2009).

A fast life history orientation (deriving in part from scarcity and unpredictability) is seen as promoting sexual precocity, impulsiveness, aggressiveness, and less investment in prosociality (Del Giudice et al., 2011). These properties reflect investment in mating and reproduction rather than in accumulation of forms of social capital that might require more time to yield reproductive pay-offs (Wolf et al., 2007; Ellis et al., 2009; Griskevicius et al., 2013). It has also been suggested that life history orientations are reflected in personality (Réale et al., 2010). A good deal of research has linked personality variables to other variables suggested by the life history framework (Del Giudice, 2014), but less attention has been devoted to linking either of these sets of variables to environmental conditions that might influence the development of one or the other life history strategy.

There are links from early adversity to problematic social behaviors of various sorts, including psychopathic traits (Glenn et al., 2011), impulsive delay discounting, indices of sexual risk-taking (Simpson et al., 2012), and financial risk-taking (Griskevicius et al., 2013). Early adversity has been related to aggressiveness (Loeber and Hay, 1997) and to men’s exploitation of interaction partners via endorsement of a “code of honor” involving preoccupation with personal honor and an orientation toward revenge rather than forgiveness (McCullough et al., 2013). Early adversity has been linked to a pattern of social difficulties including job loss, and financial and legal problems (Rosenman and Rodgers, 2006). Taken together, a good deal of research suggests that aggression and at least some aspects of impulsivity are shaped by early adversity.

On the other hand, little research has examined relations of early adversity to traits beyond aggression and these forms of impulsivity (though Rosenman and Rodgers, 2006, examined relations with the Eysenck Personality Questionnaire, finding only an association with neuroticism). The study reported here examined additional personality qualities as possible correlates of early adversity, drawing loosely on the theoretical views just outlined. We examined investment in interpersonal relations directly, via agreeableness—the broad tendency to care about, and make effort toward, maintaining positive relations with other people—and interdependence, which reflects trust of others. To replicate earlier findings, we also measured anger-proneness, hostility, and aggressiveness. All of these traits relate to investment (or lack thereof) in relationships characterized by mutual trust, concern, and respect.

Three additional measures we examined pertain to aspirations for success in extrinsic aspects of life. This was based on the reasoning that fast life histories, fostered by early life adversity, are likely dominated by extrinsic paths to status or social dominance, rather than developing status by long-term cultivation of relationships. One scale included is a measure of two plainly extrinsic motives: endorsement of the adoption of goals to attain popular fame and wealth. Another measure relevant to this theme assesses authentic and hubristic pride. Authentic pride is generally seen as deriving from efforts toward attainment of intrinsic goals, whereas hubristic pride is thought to result from seeking other people’s admiration via extrinsic status. Hubristic pride has been linked to a hierarchical dominance orientation in which status depends on social validation (Cheng et al., 2010); this orientation, in turn, relates to extrinsic goal pursuit (Duriez et al., 2007). We also included a scale that focuses on purpose and investment in life; its items refer to intrinsic engagement without reference to extrinsic reward, which should be diminished by early life adversity.

Finally, as noted above, there is evidence that early adversity is related to certain forms of impulsivity, such as sexual risk-taking and preference for immediate reward. Here we examined whether early adversity relates more generally to other trait self-reports bearing on impulsiveness, including the Self-control Scale (Tangney et al., 2004); two subscales from the UPPS Impulsive Behavior scale (Whiteside and Lynam, 2001), which assesses impulsiveness within the five-factor personality model; and the positive urgency measure (PUM; Cyders et al., 2007), which assesses impulsive reactivity to positive emotions.

In any retrospective study, there is concern about the accuracy of reporting on early life experiences (Lewinsohn and Rosenbaum, 1987), although there also is evidence in support of the accuracy of retrospective reporting (Brewin et al., 1993; Hardt and Rutter, 2004). One issue in this regard is whether the person’s current emotional state biases recall of past events, particularly events of a stressful nature. As a partial control for this problem, we used current depressive symptoms to control for the well-known tendency for general negative affectivity to confound the associations between self-reports of negative life events and self-reports of other self-relevant traits (e.g., see Watson and Pennebaker, 1989). If current depressive symptoms bias recall, then controlling for those symptoms should remove associations between reported adversity and other variables.

In sum, this study examined associations of self-reported early life adversity with a variety of personality qualities that are frequently referenced, directly or by strong implication, in existing theories.

## MATERIALS AND METHODS

### PARTICIPANTS

Participants were undergraduates at the University of Miami, who participated in partial fulfillment of a course requirement. Not all participants completed all measures; thus, Ns vary across measures (the largest was 375). The mean age of the full sample (*N* = 368; 240 females) was 18.71 years. Self-reported ethnicity was 57.1% non-Hispanic white, 24.1% Hispanic, 7.5% Asian, 4.5% African American, 2.3% Caribbean, and 4.5% other. Participants completed some or all of the measures described below, either in large group sessions at the start of the semester or in smaller groups several weeks later. There were no eligibility criteria for participation, so the sample reflects the normal range of the personality qualities assessed.

### CHILDHOOD ADVERSITY

Childhood adversity was assessed retrospectively by a self-report measure called risky families (Taylor et al., 2004). This 13-item measure was adapted by Taylor et al. (2004) from an earlier scale by Felitti et al. (1998), which was designed to assess the relation of family stress to mental and physical health outcomes in adulthood. The measure has been validated against clinical interviews conducted and coded by trained clinical interviewers (Taylor et al., 2004). It has also been found in two previous studies to potentiate genetic effects that are known to be sensitive to early life adversity (Taylor et al., 2006; Carver et al., 2011).

The risky families scale requires respondents to rate 13 aspects of their early family environment on five-point scales ranging from 1 (“not at all”) to 5 (“very often” or “very much”). Items assess the extent to which the respondent had felt loved and cared for; was insulted, put down, sworn at, or made to feel threatened; was shown physical affection; was pushed, grabbed, shoved, or slapped; was verbally abused; was physically abused; observed quarreling or shouting between parents; observed violence or aggression between family members; lived with a substance abuser; lived in a well-organized, well-managed household; and family members knew what the child was doing. Positively framed items are reverse-coded, and item responses averaged. Range, mean, SD, and alpha reliability for this and other measures are in **Table 1**.

**Table 1:** Descriptive statistics on self-reports administered.

Measure	Range	Mean (SD)	Alpha
Risky families	1.00–4.23	1.82 (0.58)	0.82
Agreeableness	1.92–4.75	3.73 (0.60)	0.78
Independence	2.00–5.00	3.80 (0.60)	0.69
Interdependence	2.00–5.00	3.71 (0.60)	0.71
Life engagement	1.17–5.00	4.24 (0.76)	0.85
Popular fame	1.00–5.00	1.85 (0.84)	0.81
Financial success	1.00–5.00	2.63 (1.15)	0.71
Authentic pride	1.00–5.00	3.42 (0.79)	0.91
Hubristic pride	1.00–3.14	1.52 (1.50)	0.80
Self-control	1.15–5.00	3.24 (0.70)	0.82
Urgency	1.00–5.00	2.78 (0.90)	0.88
Perseverance	1.00–5.00	3.98 (0.66)	0.87
Positive urgency	1.00–4.43	2.26 (0.85)	0.81
Anger	1.00–5.00	2.40 (0.97)	0.67
Hostility	1.00–5.00	2.76 (0.90)	0.61
Verbal aggression	1.00–5.00	2.78 (0.97)	0.75
Physical aggression	1.00–5.00	2.15 (1.03)	0.67
Beck Depression Inventory	0.00–40.00	8.40 (7.83)	0.87


### PERSONALITY-RELATED MEASURES

#### NEO five-factor inventory agreeableness

The NEO-FFI (Costa and McCrae, 1985) contains 60 items forming five scales assessing broad personality traits of the five-factor model of personality. A 1–5 scale is used, with labels ranging from “strongly disagree” to “strongly agree.” In this study, only the 12-item scale for agreeableness was administered. Sample items include “I respect others’ feelings,” “I take others’ interests into account,” and “I am willing to make compromises.” After appropriate item reversals, responses were averaged.

#### Independence–interdependence

The self-construal scale (Singelis, 1994) is a measure of the (separate) tendencies to be independent (12 items, e.g., “Being able to take care of myself is a primary concern for me”) and interdependent with others (12 items, e.g., “It is important for me to maintain harmony within my group”). Evidence of the validity of this measure is reviewed by Singelis (1994). Responses in this study were made on a 1–5 scale, from “strongly agree” to “strongly disagree.” Responses were averaged for each scale.

#### Life engagement test (LET)

The LET (Scheier et al., 2006) assesses the sense of purpose in life: the extent to which a person’s life includes valued activities [six items, e.g., “I value my activities a lot,” “Most of what I do seems trivial and unimportant to me” (reversed)]. This measure has been shown to have adequate psychometric properties (see Scheier et al., 2006). Respondents responded on a 1–5 scale, and responses were averaged after appropriate reversals.

#### Willingly Approached Set of Statistically Unlikely Pursuits (WASSUP)

The WASSUP (Johnson and Carver, 2006; Johnson et al., 2009) assesses the tendency to set implausibly high goals. This study incorporated only the subscales of popular fame (seven items, e.g., “You will be famous”) and Financial Success (four items, e.g., “You will run a Fortune 500 company”). Participants were asked to indicate how likely is it that they would set this goal for themselves, on a five-point scale (1 = “NO CHANCE I will set this goal for myself,” 2 = “Slight chance I will set this goal for myself,” 3 = “Moderate chance I will set this goal for myself,” 4 = “Very good chance I will set this goal for myself,” 5 = “Definitely WILL set this goal for myself”).

#### Pride

The authentic and hubristic pride scales (Tracy and Robins, 2007) use self-referent words and phrases to measure authentic pride (seven items, e.g., “like I am achieving,” “fulfilled,” “productive”) and hubristic pride (seven items, e.g., “arrogant,” “conceited,” “smug”). Respondents indicated the extent to which each item represents them, on a 1–5 scale.

#### Self-control scale

The self-control scale (Tangney et al., 2004) assesses overall tendencies toward self-control (e.g., “I am good at resisting temptation”). Self-control measured by this instrument predicts higher grade point average, better adjustment, less alcohol abuse, and better interpersonal skills (Tangney et al., 2004). The authors also reported a brief version (13 items), which was strongly related to the full measure (*r*s = 0.93 and 0.92; Tangney et al., 2004). That abbreviated scale was used here. Respondents rated their agreement to items on a 1–5 scale and responses were averaged.

#### Urgency and perseverance

The UPPS impulsive behavior scale (Whiteside and Lynam, 2001) assesses impulsive tendencies within the framework of the five-factor model of personality. Its subscales reflect distinct processes that might lead people to act without regard for potential adverse consequences, each deriving from one of the five personality factors. Two UPPS subscales were administered. Urgency is the tendency to experience strong impulses; about half the items indicate that the impulses either follow from or lead to negative affect (e.g., “When I am upset I often act without thinking”), the rest do not specify negative valence (e.g., “It is hard for me to resist acting on my feelings”). Perseverance assesses the ability to stay focused on difficult or tedious tasks (e.g., “I am a productive person who always gets the job done”). Due to response burden (sessions included other measures not addressed here), we used only 12 items from the urgency scale and 10 items from lack of perseverance (Carver et al., 2011). Items were rated from 1 to 5, and responses were averaged.

#### Positive urgency measure

The PUM (Cyders et al., 2007) assesses the tendency to act recklessly or inappropriately when experiencing positive emotions (Cyders and Smith, 2008). This measure has predicted a variety of risky behaviors such as vandalism (Cyders et al., 2007), high alcohol consumption per sitting (Cyders et al., 2009), and impulsive behavior on a laboratory task after a mood induction (Cyders et al., 2010). Positive urgency is moderately related to UPPS Urgency (*r* = 0.37), but two studies have found it to predict outcomes through different pathways than UPPS urgency (Cyders et al., 2007). We used seven items from this scale (e.g., “I tend to act without thinking when I am really excited”). Items were rated from 1 to 5, and responses were averaged.

#### Aggression questionnaire short form (AQ-SF)

The AQ (Buss and Perry, 1992) measures trait anger and aggression. Bryant and Smith (2001) shortened it, reducing overlap among the four subscales: anger (three items, e.g., “I have trouble controlling my temper”), Hostility (three items, e.g., “I wonder why I am so bitter about things”), verbal aggression (three items, e.g., “I often find myself disagreeing with people”), and physical aggression (three items, e.g., “I have threatened people I know”). The AQ-SF subscales have been found to correlate well with vignette measures of anger, hostility, verbal aggression, physical aggression, and with measures of emotional instability and alcohol consumption (Tremblay and Ewart, 2005). Ratings here were made on a 1–5 scale.

#### Depression symptoms

Current symptoms of depression were assessed using the Beck Depression Inventory (BDI, Beck and Steer, 1993). The BDI consists of 21 self-report items concerning affective, cognitive, and somatic symptoms of depression. Responses are summed. The scale has adequate internal consistency (in this sample, α = 0.87), is highly correlated with other measures of depression, and highly correlated with risk variables for depression (Gould, 1982; Knight, 1984; Scogin et al., 1984). The BDI is highly correlated with self-report measures of negative affectivity (Watson et al., 1988), and thus is adequate for controlling the possibility that reports of early life adversity and personality are confounded by negative affectivity.

### DATA REDUCTION

The scales other than risky families and BDI were reduced to a smaller number of latent variables by factor analysis, using both exploratory and confirmatory methods. We first conducted a purely exploratory analysis (with oblique rotation to permit correlated factors) to identify variables to serve as anchors for subsequent analysis (the highest loading variable on each factor with eigenvalue greater than 1). This analysis necessarily omitted any participant with missing data. This was followed by an exploratory analysis conducted within the framework of confirmatory factor analysis using Mplus version 7 (Muthén and Muthén, 1998–2012), which allowed the use of full information maximum likelihood estimation, thereby retaining all data from all participants (Jöreskog, 1969; Muthén and Muthén, 2009). We used exploratory rather than confirmatory analysis here because we had hypotheses about relations of the various scales to risk, but not necessarily to each other. The four anchor items were fixed at 0 on all factors for which they were not anchors, all other loadings were free to vary, and factor variances were fixed at 1. (We also tested a three-factor model, which performed significantly worse than the four-factor model, comparison *p* < 0.001).

Without modifications, the four-factor model did not fit the data well, χ^2^(62) = 197.726, *p* < 0.001, RMSEA = 0.076, 95% CI(0.065, 0.088), CFI = 0.898, SRMR = 0.049. To improve model fit, residual variances were allowed to correlate among the following observed variables: Persistence with Self-control, urgency with self-control, positive urgency with urgency, financial success with popular fame, and anger with hostility. The chi-square for this model was still significant, χ^2^(56) = 78.572, *p* = 0.025, but it met the recommended cut-off criteria for three other fit indices, RMSEA = 0.033, 90% CI(0.012, 0.049), CFI = 0.983, SRMR = 0.034. This modified model was retained for analyzing the relationships between our predictors and the latent variable outcomes.

**FIGURE 1:**
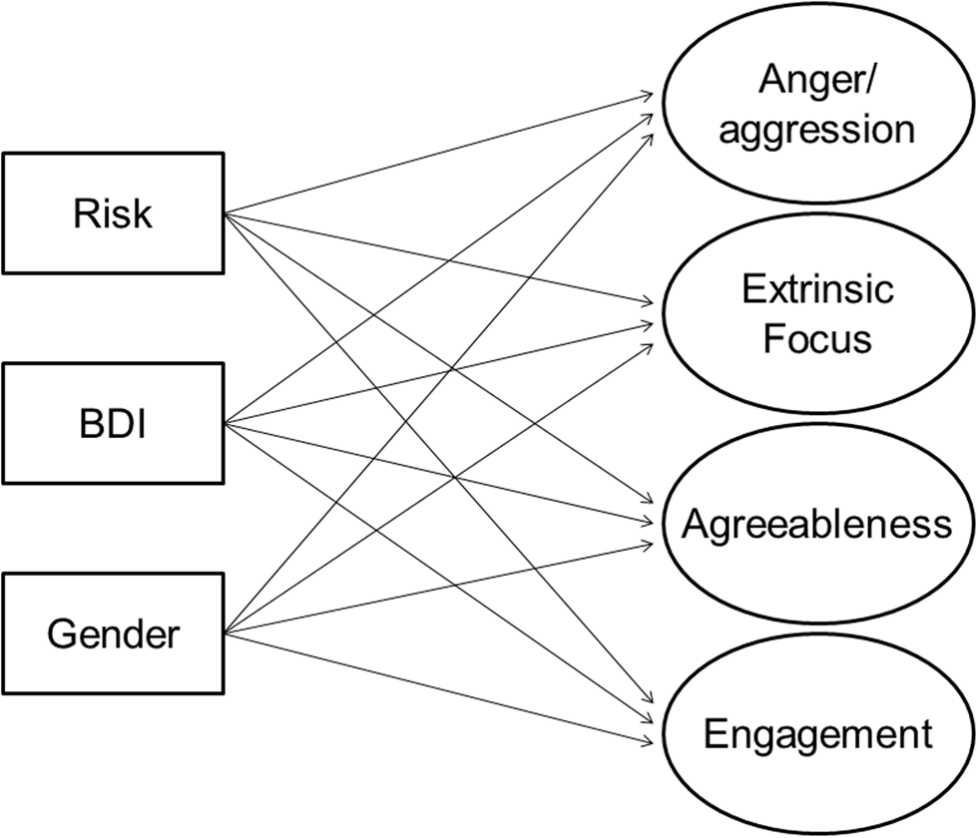
**Structural model tested, in which four latent personality variables are predicted by risk, Beck Depression Inventory (BDI) scores, and gender**.

Loadings from the final model are in **Table 2** along with Ns for the subsamples that completed each measure. The latent variables were labeled anger/aggression, extrinsic focus, agreeableness, and engagement. These latent variables were used as outcome variables in the analyses reported below. We first examined bivariate correlations among the risky families scale, the four latent personality variables, and BDI. The main analysis was a structural model in which all four latent personality variables were predicted from Risk, BDI, and gender as an additional control variable (**Figure 1** shows the model tested). Finally, we tested the generality of that model across gender.

**Table 2:** Standardized loadings for personality scales, from exploratory factor analysis within confirmatory framework, using full information maximum likelihood estimation.

Measure	*n*	1	2	3	4
		Anger/aggression	Extrinsic focus	Agreeableness	Engagement
AQ anger	349	1.72**	-0.67	0.55	-0.11
AQ physical aggression	349	0.68***	0 ^†^	0 ^†^	0 ^†^
AQ hostility	350	1.19**	-0.52	0.43	-0.26
AQ verbal aggression	349	0.75**	0.07	-0.39***	0.09
Urgency	360	0.67**	-0.32	0.17	-0.29**
Financial success	367	0 ^†^	0.44***	0 ^†^	0 ^†^
Popular fame	367	-0.07	0.44**	-0.10	-0.07
Hubristic pride	150	-0.45	1.09*	-0.11	-0.25
Agreeableness	344	0.05	-0.60*	0.71***	0.13
Interdependence	217	0 ^†^	0 ^†^	0.56***	0 ^†^
Authentic pride	150	-0.36	0.48*	-0.02	0.62***
Perseverance	366	-0.15	0.12	-0.11	0.49***
Self-control	368	-0.22	-0.00	-0.02	0.36***
Positive urgency	334	-0.01	0.43*	-0.09	-0.41***
Life engagement	344	0 ^†^	0 ^†^	0 ^†^	0.76***
Independence	217	-0.32	0.59*	-0.38*	0.44***

*p < 0.05, **p < 0.01, ***p < 0.001, ^†^ loading fixed to 0.

## RESULTS

Correlations among variables are in **Table 3**. Of greatest interest are those in the first column, between risky families and the other measures. As can be seen, reports of early adversity related to lower levels of agreeableness and engagement and to higher levels of anger/aggression and extrinsic focus. Reports of early adversity also related to higher levels of depression symptoms.

**Table 3:** Correlation estimates between risky family scores, personality latent variables, and depression symptoms.

Measure	Risk	Anger/aggression	Extrinsic focus	Agreeableness	Engagement
Risk	-				
Anger/aggression	0.34***	-			
Extrinsic focus	0.21**	0.74***	-		
Agreeableness	-0.31***	-0.28***	0.21**	-	
Engagement	-0.20**	-0.15**	0.05	0.32***	-
Beck Depression Inventory	0.32***	0.37***	0.12*	-0.16**	-0.53***

*p < 0.05, **p < 0.01, ***p < 0.001.

The association with depression symptoms is of potential significance in its own right. However, for present purposes the most important issue is the potential argument that associations of early adversity with other variables are simply an artifact of a general overall negativity which is reflected in this association as well (Lewinsohn and Rosenbaum, 1987; Watson and Pennebaker, 1989). For this reason, the main analysis was a structural model in which all four latent personality variables were predicted from early adversity, BDI, and gender as an additional control variable (**Figure 1**). The chi-square for this model was significant, χ^2^(92) = 152.009, *p* < 0.001, but recommended criteria for three other fit indices were met, RMSEA = 0.042, 90% CI(0.030, 0.053), CFI = 0.963, SRMR = 0.042. The standardized regression coefficients for the predictors from this analysis are in **Table 4**, along with the overall *R*-square for each outcome variable. BDI scores were uniquely related to two of the personality factors. However, significant unique associations remained between the risky families scale and all personality factors except for engagement.

**Table 4:** Standardized regression coefficients (betas) and *t* values for self-reported childhood adversity (Risk), gender, and concurrent depression scores (BDI), from a structural equation model predicting four latent personality factors (*n* = 375).

		Anger/aggression	Extrinsic focus	Agreeableness	Engagement
Risk	Beta	0.23	0.22	-0.29	-0.02
	*t*	3.88***	2.17*	3.64***	0.34
Gender	Beta	0.38	0.58	-0.01	-0.23
	*t*	3.92***	5.05***	0.09	3.75***
BDI	Beta	0.31	0.12	-0.10	-0.55
	*t*	4.65***	1.09	0.80	10.17***
*R*-square		0.31	0.39	0.11	0.33

* *p* < 0.05, *** *p* < 0.001.

**FIGURE 2:**
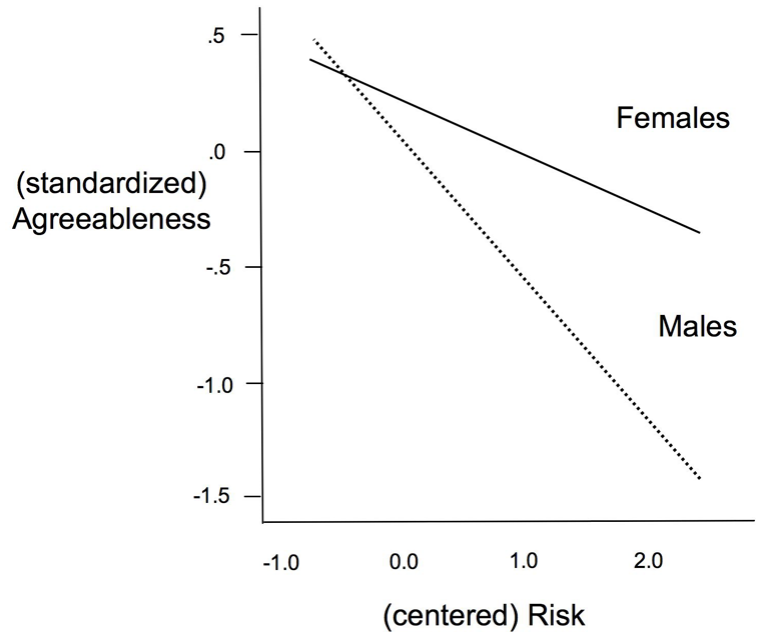
**Interaction between gender and risk in prediction of agreeableness latent variable**.

One further analysis tested for the possibility that early adversity might relate to the personality factors differently by gender, by adding a gender-by-risk interaction to the prediction of each latent variable. The chi-square for this model was significant, χ^2^(104) = 170.255, *p* < 0.001, but the model met criteria for three other fit indices, RMSEA = 0.041, 90% CI(0.03, 0.052), CFI = 0.959, SRMR = 0.041. One significant interaction emerged, for agreeableness, β = -0.46, *t*(370) = 1.99, *p* = 0.046. The form of this interaction (**Figure 2**) was that adversity related to lower agreeableness among both men and women, but the association was stronger (by simple slope analysis) for males, *p* < 0.001, than for females, *p* = 0.051. No other interaction approached significance.

## DISCUSSION

This study examined how an adverse childhood environment (assessed by retrospective self-reports) might be reflected in a number of adulthood traits. As noted in the introduction, Belsky et al. (1991) have proposed that children use early life experiences to create a model of the availability and predictability of resources, and the nature of interpersonal relations. This perspective suggests that early adversity can create expectations that resources will be scarce and the implicit view that relationships are disposable, yielding an opportunistic orientation to relationships (Belsky, 2012). Others have suggested that aspects of early adversity set the stage for a fast life history orientation, promoting competitive, aggressive pursuit of resources with little investment in longer-term relationship building. Little research, however, has directly examined the link of early adversity to personality traits suggested by these views beyond aggression and specific forms of impulsivity.

The study reported here examined a somewhat broader range of personality traits, with particular emphasis on traits reflecting investment in relationships, on the one hand, and commitment to extrinsic goals, on the other. We began by distilling the measures to four factors, which we labeled anger/aggression, extrinsic focus, agreeableness, and engagement. Early adversity was related to greater anger/aggression and to lower agreeableness, consistent with a picture in which early adversity fosters anger, hostility, and aggression and little investment in relationships (see Del Giudice, 2014). It is of interest that these associations point directly to the big-five trait of agreeableness, a measure of which was central to the agreeableness factor. Given definition of agreeableness as the broad tendency to care about, and to make effort toward, maintaining positive relations with other people, involvement of this trait appears consistent with the thrust of the theoretical argument made by Belsky et al. (1991) and Belsky (2012).

Also consistent with theory, persons who reported early adversity displayed a desire to seek out social recognition and status, reflected in a positive association with extrinsic focus, a variable that was composed largely of hubristic pride and the intention to pursue popular fame and wealth. This competitive pursuit of resources is in line with an emphasis on superficialities and the seeking of social position by means other than relationship building. This quality does not fit readily into a big-five framework, though it does seem to be conceptually related to the honesty-humility factor in the six-factor HEXACO framework (Ashton and Lee, 2007, 2008).

The associations just described all remained significant when a control was instituted for concurrent depressive symptoms, though an association with the engagement factor did not. It is arguable that the control for depressive symptoms is overly conservative, inasmuch as early adversity has the potential to increase vulnerability to depression as well as to affect the other properties under investigation. Nonetheless, the pattern militates strongly against the argument that associations of risk with agreeableness and anger/aggression were a byproduct of a general negativity (cf. Watson and Pennebaker, 1989). Indeed, the fact that risk did not uniquely relate to engagement (another clearly positive property of personality) further weakens that alternative argument. The associations were not general to all positive qualities, but were specific to social ones.

This study has several important limitations, including the fact that effect sizes were modest. It used a retrospective account of childhood experiences, albeit one that has been validated against individual interviews (Taylor et al., 2004) and has been found to potentiate a genetic effect that is independently known to be responsive to early adversity (Taylor et al., 2006; Carver et al., 2011). It used a convenience sample that was relatively well-educated and young. The levels of early adversity reported were relatively low and the measure of early adversity was broad, not lending itself to testing of more focused hypotheses pertaining to either Belsky’s (2012) theory or life history theory. Future studies would do well to sample participants who have been known to be exposed to early childhood adversity, as has been done fruitfully in recent studies of orphans (e.g., Hostinar et al., 2012).

Finally, given the cross-sectional nature of the assessment, it is not possible to make inferences about causal influences. A variety of third-variable interpretations can be raised. These include the possibility that the core personality trait behind the associations was in place during childhood and rendered the children more versus less aware of interpersonal difficulty in the family (or more versus less likely to impose that interpretation on events). It is also possible that early adversity covaries with genetic predispositions to the personality traits examined here, though there is at least some evidence that the effects of early family chaos are transmitted environmentally (Jaffee et al., 2012).

Despite these cautions, and although the evidence is suggestive rather than firm, the pattern of results is consistent with a picture in which early family adversity hardens people and turns them more competitive and less committed to positive social engagement. This pattern is also consistent with previous findings linking early adversity with psychopathic traits (Glenn et al., 2011) and exploitation of others (McCullough et al., 2013). The findings presented here, tentative though they certainly are, suggest that personality effects of adverse early experience warrant further exploration.

## Conflict of Interest Statement

The authors declare that the research was conducted in the absence of any commercial or financial relationships that could be construed as a potential conflict of interest.
